# Social protection in areas vulnerable to tuberculosis: a mixed methods study in São Luís, Maranhão

**DOI:** 10.1590/0034-7167-2023-0428

**Published:** 2024-06-17

**Authors:** Francisca Bruna Arruda Aragão, Mellina Yamamura Calori, Thais Zamboni Berra, Antônio Carlos Vieira Ramos, Ethel Leonor Noia Maciel, José Henrique da Silva Cunha, Larissa Barros de Souza, Marcelino Santos, Ricardo Alexandre Arcêncio, Regina Célia Fiorati

**Affiliations:** I Universidade de São Paulo. Ribeirão Preto, São Paulo, Brazil; II Universidade Federal de São Carlos. São Carlos, São Paulo, Brazil; III Universidade Federal do Espírito Santo. Vitória, Espírito Santo, Brazil; IV Universidade Federal do Maranhão. Imperatriz, Maranhã, Brazil

**Keywords:** Social Determinants of Health, Public Policy, Social Program, Tuberculosis, Social Vulnerability, Determinantes Sociales de la Salud, Política Pública, Programa Social, Tuberculosis, Vulnerabilidad Social

## Abstract

**Objectives:**

to analyze the risk areas for tuberculosis and the influences of social protection on the development of treatment for the disease in the municipality of São Luís, Maranhão.

**Methods:**

this is explanatory sequential mixed method research. In the quantitative phase, the data were obtained from the Notifiable Diseases Information System from 2010 to 2019, with georeferencing being carried out to identify areas vulnerable to tuberculosis. In the qualitative phase, semi-structured interviews were carried out with individuals who received social benefits.

**Results:**

7,381 cases were geocoded, and, from the purely spatial scanning analysis, it was possible to identify 13 spatial clusters of risk. As for the interviews, there was a positive relationship between patient improvement and receiving benefits.

**Conclusions:**

geographic space and social determinants are relevant for reorienting monitoring actions for the conditions that generate the health-disease process.

## RESUMEN

**Objetivos:**

analizar las áreas de riesgo para la tuberculosis y las influencias de la protección social en el desarrollo del tratamiento de la enfermedad en el municipio de São Luís, Maranhão.

**Métodos:**

se trata de una investigación explicativa de método mixto secuencial. En la fase cuantitativa, los datos se obtuvieron del Sistema de Información de Enfermedades de Declaración Obligatoria del 2010 al 2019, realizándose georreferenciación para identificar áreas vulnerables a la tuberculosis. En la fase cualitativa se realizaron entrevistas semiestructuradas a personas que recibían beneficios sociales.

**Resultados:**

se geocodificaron 7.381 casos y, a partir del análisis de escaneo puramente espacial, fue posible identificar 13 grupos espaciales de riesgo. En cuanto a las entrevistas, hubo una relación positiva entre la mejora del paciente y la recepción de beneficios.

**Conclusiones:**

el espacio geográfico y los determinantes sociales son relevantes para reorientar las acciones de seguimiento de las condiciones que generan el proceso salud-enfermedad.

## INTRODUCTION

Until the emergence of COVID-19, a disease caused by Severe Acute Respiratory Syndrome Coronavirus 2 (SARS-CoV-2), tuberculosis (TB) was the infectious disease that killed the most in the world, however it currently ranks second. Furthermore, it leads the rates among infectious diseases in developing countries, mainly affecting countries and populations in situations of social vulnerability. It is estimated that approximately 1/3 of the population is infected by the *Mycobacterium tuberculosis* complex, thus making it even more difficult to control the disease, mainly in groups and/or populations that are at greater risk of illness, influenced by conditions and Social Determinants of Health (SDH)^(1)^.

Brazil is on the list of 30 countries that account for 87% of the TB burden, ranking 20^th^ worldwide, along with 15 other countries, accounting for 93% of TB notifications in the world, which constitutes a major challenge for managers and healthcare professionals^(1)^. The disease is associated with social, political and cultural determinant, as well as the population’s living conditions^(2)^, determined by biological factors, such as malnutrition, Human Immunodeficiency Virus (HIV) infection, age group, and social factors, such as unhealthy housing, high demographic density, inadequate working conditions and lack of access to health services^(3)^.

Considering the above, the relationship with social, structural and intermediary determinants, and social protection policies is essential to guarantee improvements in social conditions and, thus, a better quality of life for people affected by the disease. Therefore, in the current study, the theoretical basis used was the SDH model from the World Health Organization (WHO) SDH Commission^(4)^. In this context, findings in the literature indicate an increase in cure rates in countries that include a person-centered care approach and offer social support to persons with TB and their family, such as transportation vouchers, market baskets and financial resources^(5)^.

It is worth highlighting that studies show a drop in a person’s quality of life after starting treatment due to the side effects of medication, making i is essential to use pharmacological and non-pharmacological strategies to reduce these effects, and social support makes all the difference in this clinical management^(5)^.

Another issue refers to catastrophic costs, causing persons with TB to have many financial expenses with treatment, due to frequent visits to health services, interruptions in working days, transport costs, food expenses outside the home, among others^(3)^.

In this way, the need for greater involvement between healthcare professionals, managers and the scientific community is evident, in order to implement operational and research measures that are relevant to the knowledge of social conditions related to the health-disease process and measures and strategies to mitigate TB^(3)^.

Thus, in the literature, there have been studies intended to understand TB dynamics in territories, through the use of geoepidemiology, through the Geographic Information System (GIS); however, there is still a lack of studies that demonstrate the coverage of social protection programs in these areas.

To this end, spatial analysis is observed as a technique for collecting and processing information in a given geographic space. When used in the health sector, it allows mapping health problems and risk assessment, contributing to identifying vulnerable groups, risk areas and priorities, supporting the development of targeted and comprehensive public health interventions and policies.

## OBJECTIVES

To analyze the risk areas for TB and the influences of social protection on the development of treatment for the disease in the municipality of São Luís, Maranhão.

## METHODS

### Ethical aspects

The study in the quantitative phase was exempt from the Informed Consent Form (ICF), as the data search was carried out using the Notifiable Diseases Information System (SINAN - *Sistema de Informação de Agravos de Notificação*), available at https://portalsinan.saude.gov.br/tuberculose in a universal and integrated way. However, in the qualitative stage, the study was conducted in accordance with national and international ethical guidelines. It was approved by the Research Ethics Committee of the *Escola de Enfermagem de Ribeirão Preto* (EERP). Hence, the ICF was obtained from all individuals involved in the study through a written document, one copy being delivered to participants and the other to the researcher. To guarantee participant anonymity, the letter I (interviewed) was used, followed by the sequential number of the interviews.

### Study design

This is mixed methods research, combining quantitative and qualitative approaches, characterized by a sequential explanatory design. This is an explanatory design because the quantitative stage precedes the qualitative stage in two distinct but interactive and sequenced phases^(6)^. The study follows the standards in accordance with Preferred Reporting Items for Systematic Review and Meta-Analysis Protocols (PRISMA). It was carried out from 2010 to 2019 in the municipality of São Luís, Maranhão, Brazil, with seven health districts.

### Population and sample

The study population was made up of individuals over 18 years of age, diagnosed and undergoing treatment for TB and who received some benefit from Social Protection identified in risk areas.

Inclusion criteria included patients diagnosed with TB who received any social, federal, state or local benefit aged 18 years or older, residing in the urban area of the city, whose diagnosis is described in the International Classification of Diseases version 10 – (ICD10), with corresponding codes from A15.0 to A19.0. Exclusion criteria included notifications of diagnosed cases that had an address in other municipalities in the state of Maranhão and/or without a full address on the notification form, cases of repeated data entry, age under 18 years, patients who were not included in social benefit programs.

In the quantitative stage, data collection was carried out using the SINAN notification form, which was used to map risk areas for TB. The form should be duly completed for cases identified at least one year old. For inclusion criteria, TB cases diagnosed between January 1, 2010 and December 31, 2019 were considered. It is worth highlighting that data from SINAN notification forms for the years 2010 to 2019 were used. The municipality of São Luís only had seven health districts.

### Analysis of results, and statistics

In the quantitative stage, data collection was carried out using the SINAN form. Confirmed cases of TB residing in São Luís were considered and, when a person affected by TB was notified more than once, only the most current record was considered.

From the notification form, the residential address of each case was collected and, subsequently, georeferencing was carried out, based on geographic coordinates (latitude and longitude) obtained using Google Earth™.

To detect risk clusters, the purely spatial scanning statistics technique was applied^(7)^. In the analysis, clusters are identified through circular windows with variable radius around the centroid of each census tract of the municipality under analysis, where the expected number of cases within this circle is calculated. Thus, the Relative Risk (RR) of each cluster was determined, indicating the intensity of occurrence of concomitant TB cases in the municipality analyzed. For interpretation, when the RR of a cluster is equal to 1 (RR=1), there is strong evidence that there are no clusters; if less than 1 (RR<1), tending to zero, it will be low risk (or protection); above 1 (RR>1), it will be a risk area. Clusters with a p-value <0.05 (α=5%) were considered significant.

Georeferencing and creation of maps were carried out using QGIS 3.16.2 software. Scan analysis was performed using SaTScan™ software version 9.2. Interviewee characterization was carried out using Stata software version 13.0, and for content analysis, ATLAS program version 9 was used.

The qualitative phase was carried out based on risk areas identified in the previous stage. This stage was conducted based on the Consolidated criteria for Reporting Qualitative research (COREQ), with monitoring of quantitative results that were used to refine the qualitative questions. Afterwards, interviews were carried out guided by semi-structured scripts addressing analytical categories that raise questions about knowledge and practices about TB, receipt of social assistance benefits, importance of these benefits for the patient and their influence on treatment, aimed at people diagnosed and identified from the medical records of patients undergoing treatment in Basic Health Units (BHU) and in Family Health Strategy (FHS), located in regions with the highest risk for TB and previously identified in the quantitative stage.

It is noteworthy that, at this stage, only patients diagnosed with TB who received any federal, state or local social benefit were interviewed. For this, an interview script was created with TB patients in São Luís, Maranhão.

After identifying potential participants, these people were contacted by the researcher to present the study. After this moment, the qualitative questions that were included in the interview script were refined.

Data from medical records and individual statements were kept confidential; the recordings were made in audio to maintain the information as it was actually reported; and each interview lasted approximately one hour, being available to participants and kept under the responsibility and custody of the researcher.

It is worth mentioning that the interviews were carried out between August and October 2021. These interviews followed criteria such as scheduled date and time. Based on availability, the collection of statements took place at BHU, in previously reserved rooms. It is noteworthy that one of the 16 interviews took place at the home of one of the participants who was diagnosed with TB and, therefore, had mobility difficulties. Due to this difficulty, aiming at the well-being of this patient, the researcher went to the home, accompanied by the healthcare team and the community worker to carry out the interview.

For qualitative data analysis, the content analysis technique^(8)^ was used, unfolded in the pre-analysis, followed by text skimming of interviews, allowing the researcher to delve deeper into the collected material.

Then, they were read exhaustively, aiming for deep understanding. In the content exploration phase, the meaning cores were identified, which were systematically grouped, analyzed and reorganized, giving rise to analytical categories.

## RESULTS

**Figure 1:**
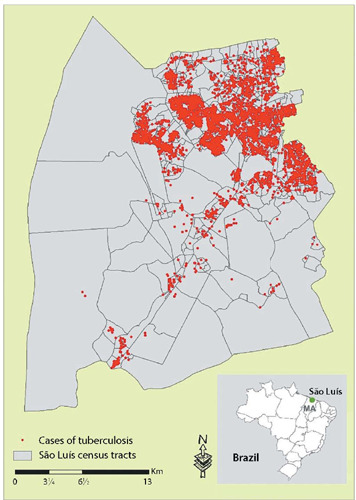
Spatial distribution of tuberculosis cases living in São Luís, Maranhão, Brazil, between 2010 and 2019

According to SINAN data, of the 7,958 TB cases reported between 2010 and 2019, 7,381 (92.7%) were geocoded. According to the sociodemographic profile, the majority represented male individuals (67.92%) aged 20–29 years (27.07%) and 30–39 years (23.67%). The largest portion of cases declared themselves brown (50.11%), having one to eight years of education (47.40%). A small portion claimed to receive some type of government aid (4.39%) (Table 1). Figure 1 represents the cases distributed in the spatial territory of the municipality, in which a heterogeneous distribution of cases is noted.

**Table 1:** Characterization of spatial risk clusters for tuberculosis cases in São Luís, Maranhão, Brazil, from 2010 to 2019

Identification	Number of tracts	Total cases	Population
1 *Centro de Saúde de Pedrinhas I*	1	556	1,300
2 *USF Vila Sarney, Centro de Saúde Thales Ribeiro, Centro de Saúde Tibiri*	1	25	220
3 *Centro de Saúde Fabriciana Moraes*	1	61	1,525
4 *USF Olímpica III*	2	13	388
5 *USF Vila Sarney*	2	101	3,622
6 *USF Santa Efigênia*	1	33	1,183
7 *Centro de Saúde Radional*	1	26	992
8 *Centro de Saúde São Raimundo*	1	25	1,042
9 *USF Olímpica com USF Olímpica II na proximidade*	1	25	1,222
10 *Centro de Saúde Yves Parga, Centro de Saúde Janaina, Centro de Saúde Laura Vasconcelos, Centro de Saúde Dr. Paulo Ramos e Centro de Saúde da Liberdade*	94	924	78,226
11 *Centro de Saúde Bairro de Fátima, Centro de Saúde do João Paulo, Centro de Saúde Dr. José Carlos Macieira, Centro de Saúde Especialidades Médicas da Vila Esperança, Centro de Saúde Antônio Guanare, Centro de Saúde Genésio Rêgo, Centro de Saúde Vila Embratel, Centro de Saúde Vila Bacanga Embrião e Centro de Saúde Itapera*	217	2,773	27,727
12 *Centro de Saúde Bairro de Fátima*	2	852	79,219
13 *Centro de Saúde Gapara com Centro de Saúde da Vila Embratel na proximidade*	2	474	44,970

The distribution, although heterogeneous, is concentrated on the coastal strip and in the extreme north and northwest of the region. In the purely spatial scanning analysis, it was possible to identify 13 spatial risk clusters for TB, presented in Table 1.

**Figure 2:**
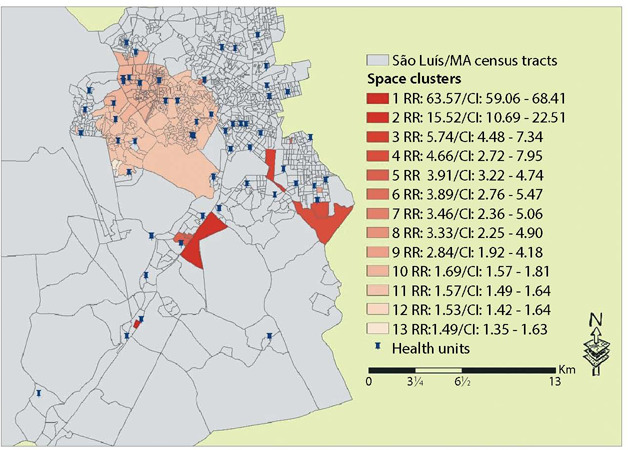
Spatial risk areas for tuberculosis according to census tracts, location of health units and relative risk of each cluster

Figure 2 shows the identified risk areas, with purely spatial scanning analysis, according to census tracts in São Luís, Maranhão.

### Qualitative step

This stage was carried out with patients from cluster 11, due to the greater number of health centers available. Thus, Bardin’s content analysis technique was used, in which 16 people were interviewed, of which 20 people did not report receiving any social assistance. After content analysis, the interviews were divided into three main analytical categories.

Regarding “Receipt of social assistance benefits”, it was found that the majority received some social assistance benefit before the disease diagnosis. Among the aids mentioned are retirement, considered as a social assistance benefit, concomitantly with Family Allowance, however, there were reports regarding the receipt of emergency aid as help in supporting their family.

*I receive Family Allowance, but now I am receiving emergency aid.* (I9)

*I receive R$89.00 per month from Family Allowance, I even registered there at* [center] *when I was in a vulnerable situation. I was unemployed, homeless, that’s why I received this aid from the Federal Government.* (I10)

*I receive my pension, it’s not much, but it helps us here at home. My retirement has already helped me, because I bought a motorcycle and paid for it with just that money. I didn’t take money from my business. It helps me.* (I14)

[...] *because I get Family Allowance here, so Family Allowance should increase a little.* (I16)

In the analytical category “Importance of the benefit”, it appears that the narratives of these patients’ lives before receiving the benefit are focused on people’s difficulties in raising children, food, housing, education and health.

*Before the benefit, I only had my wage, which is not much* [...] *so, in some months, I was missing things for the house or for the children.* (I6)

*It was difficult before, because my husband is unemployed, and I need to pay for transportation, I need to* [...] *feed myself too, since the food is a little precarious.* (I8)

*We have five people living here at home, and only my husband works, so there were days when we didn’t have much to eat as well as not being able to buy some basic items for the children, such as shoes.* (I10)

*Oh, if I didn’t receive the allowance, I wouldn’t be able to live well and my children would lack a lot of things.* (I15)

*I think we should get paid, because we can’t work, so how are we going to support ourselves?* (I5)

In relation to the analytical category “The benefit and its influence on tuberculosis treatment”, positive associations were found between social beneficiary programs and treatment duration, since their direct effects are related to the reduction of treatment abandonment due to the possibility of purchase of medications, which is associated with increased healing and, consequently, a reduction in deaths related to the disease, in addition to purchasing food for themselves and their family members.

*Yes, both for me and for other people. When we are going through difficulties and don’t even have money to buy medication, it helps us a lot.* (I1)

*It helps a lot, yes it does, I need to eat well, I need a normal diet so I can gain weight* [...] *I’ve already gained a lot! Before, my weight was not normal, it was very low*. (I3)

*Yes, there is,* [...] *as I’m not working, this money helps me buy food and fruit.* (I4)

*I believe so,* [...] *in the current crisis we are going through, there are food problems* [...] *I cut out some foods* [...] *I can eat well, but there are some patients who need to buy food to complement treatment, and this assistance could improve recovery.* (I12)

However, it was observed that three patients reported that the benefit did not produce any change, as they found the value acquired to be little.

*You can’t buy many things, but* [...] *it helps a little, you can go by.* (I2)

*No, because, as I do a side job here and there, I don’t just depend on that money, because with that money, I can’t do everything I want, you know?* (I9)

*No, it didn’t influence much, no, because R$89 is very little.* (I10)

## DISCUSSION

In this research, risk areas related to TB were identified, entering into an in-depth analysis of how social protection policies affect the advancement of treatment for this disease in the municipality of São Luís, Maranhão, where the majority represented male individuals aged between 20–29 years and 30–39 years, brown and with one to eight years of education. A portion claimed to receive some type of government aid.

Silva *et al*.^(9)^, in an epidemiological analysis of the correlation between sociodemographic factors and the incidence of TB in the Northeast region, found that the disease affects more men (63.2%), brown individuals (60.2%) and people with aged between 20 and 64 years (70.6%), supporting the findings.

Likewise, a study by Oliveira *et al*.^(10)^, analyzing the profile of TB in northeastern Brazil from 2008 to 2018, observed that the disease had a higher incidence among individuals aged 20 to 29 years, with majority in males (38,306 cases).

It is also mentioned that education is a SDH, since education influences an individual’s ability to acquire health promotion information, preventing diseases and increasing adherence to treatment^(11)^.

From 2010 to 2019, 13 spatial clusters were identified that present a high risk for the occurrence of TB in São Luís. The clusters identified are spaces favorable to presenting the highest rates of the disease, due to characteristics such as greater population concentration and intense flow of people coming from different locations. According to Silva *et al*.^(9)^, the greater number of TB cases in less favored regions is a reflection of the lack of actions linked to the search for respiratory symptoms for early detection and, also, lower demand by residents for health services in peripheral regions as well as the pronounced population agglomeration in the region, resulting in a greater number of TB cases and high incidence. In view of this, this scenario demonstrates social inequality in Brazil, since access to health resources, education, income distribution and basic sanitation is closely related to the risk of becoming ill from TB in different social strata.

In research by Asemahagn, Alene and Yimer^(12)^, carried out in Ethiopia, they found that the majority of clusters of TB cases were detected in the central (urban and surrounding areas), north and northwest of the area. The authors emphasize that these areas have large geographic coverage with unfavorable characteristics (valleys, mountainous and limited transport infrastructure), high demographic density with low income, limited access to health facilities and little community awareness about TB prevention.

It is therefore considered that the area of the studied municipality is heterogeneous and may present greater variability in the distribution of health indicators and, consequently, in TB rates. It should also be highlighted that the disease may be predominantly related to poverty, supporting the relationship between neglected disease and SDH.

Through narratives about the situation in which these families lived in the period before the benefit, it is possible to observe the multiple problems they faced and which represent obstacles to their autonomy as citizens. This situation is in line with the WHO SDH, as interviewees present vulnerabilities and social inequities, producing differentiated access to material and symbolic resources that impact people’s and populations’ health.

According to a study by Alves *et al*.^(13)^, poverty was a fundamental social determinant in explaining the cluster of risk of death from TB. The authors state that an important social determinant refers to family income, given that the results indicate that increasing this factor reduces the risk of death from TB, i.e., areas with higher income have better levels of general health and little mortality from TB illness. Thus, low income and poverty are indicators that are related, although they are not restricted exclusively to this aspect.

Public health services are essential for controlling TB, but they are insufficient in the country. Improvements in TB control services and programs can lead to improvements in care, with faster and more effective diagnosis and management of TB cases.

Furthermore, finding and treating the poorest people remains the greatest challenge, as the focus on the vulnerable population determines multiple approaches that are generally not within the standard competence of healthcare professionals in Brazil^(14)^.

In this context, the importance of benefits arising from social protection system programs for this population is observed, becoming evident throughout the study that the majority consider it essential for their survival and for TB treatment.

Orlandi *et al*.^(15)^ state that Brazil provides TB treatment free of charge in the Brazilian Health System (SUS – *Sistema Único de Saúde*), but there is evidence that patients have significant health expenses even indirectly, such as the need for transportation to go to health and food services. In the case of TB, it is estimated that these expenses compromise around 8% to 20% of patients’ annual income. Therefore, social support, with the provision of food, transportation vouchers or monetary support, can reduce the effects of the disease and help overcome some treatment barriers. In the research by the aforementioned authors, it was observed that receiving the market basket and transportation voucher was relevant for adherence to treatment as well as for the bond between patients and the healthcare team.

According to the study participants who were located in the regions that make up the risk clusters for TB, it was identified that social benefits, such as the former Family Allowance, currently called *Auxílio Brasil* (Brazil Aid), and emergency aid, were highlighted as important guarantees to combat unemployment, especially during the COVID-19 pandemic.

Oliosi *et al*.^(16)^ observed that the cure rate was 7.6% higher in the group that received Family Allowance compared to the group that did not receive it, and the proportion of patients who gave up was 7% lower in the group than in those who received the benefit. According to the authors, Family Allowance has a direct effect on the outcome of TB treatment. This is in line with the study by Carter *et al*.^(17)^, which demonstrated a substantial absolute increase in the success rate of TB treatment (7% and 11%) among those receiving Family Allowance.

It should be remembered that COVID-19 had a major impact on TB treatment, as it caused additional challenges beyond epidemiological surveillance, at the national and international levels, and in the recognition of the need for public policies aimed at reducing inequalities in access to systems health and the urgent reduction of social injustice^(18)^.

According to Maciel *et al*.^(18)^, COVID-19 and TB are different diseases, with different impacts on public health, establishing different government actions to combat them, without correct investments for control and innovation for both diseases. In Brazil, funding for TB did not reach 0.1% of the total amount allocated to science and technology in all areas at the time of the pandemic. According to the Brazilian government’s Transparency

Portal, the total amount invested in TB research over the last ten years was just over US$6 million, an amount that was further reduced during the pandemic period^(19)^.

Torres and Rabahi^(20)^ state that another immediate impact of the COVID-19 pandemic was the reduction in new diagnoses. In other words, increased TB notifications worldwide between 2017 and 2019 was followed by an 18% decrease in the interval between 2019 and 2020 (from 7.1 million to 5.8 million), in which Brazil is among the countries that contributed most to this decrease. Concomitant to this overview, there was an increase in deaths from TB in global, regional and country areas, reversing years of progress in reducing the number of deaths from the disease^(21)^.

This scenario is also demonstrated, according to Migliori *et al*.^(22)^, through the reduction in TB testing in some of the countries included in the study, such as the Philippines, Kenya and Brazil. Preventive therapy-related declines were 30% to 70% in several TB centers in Brazil, Kenya, the Philippines, and Russia. The authors also emphasize that, to contain the spread of the new coronavirus, society was advised to stay at home. However, this policy has become unfeasible in some aspects, since, in some developing countries, informal jobs are the majority: 54% in Latin America; 67% in Southeast Asia; and 86% in Africa.

Therefore, these workers were not able to have the option of staying at home and not all governments were able to provide emergency financial aid, which would allow these people to remain at home. The higher the poverty level, the greater the number of COVID-19 cases, which consequently led to a decrease in resources and awareness directed toward other diseases, such as TB^(23)^.

Horta *et al*.^(24)^, through a study with 133 Brazilian cities during the pandemic period, demonstrated that, of the 33,250 individuals surveyed, 11.8% reported that they stopped seeking care despite being sick, 17.3% did not attend routine or screening appointments and 23.9% reported one or both of these outcomes. The study also demonstrated that individuals with lower socioeconomic status and education were more likely to not seek health services in case of TB. Souza *et al*.^(25)^, based on this, stated that the SDH must be understood beyond their biomedical aspects, incorporating subjectivity of access, bonding, reception, health needs, adherence to treatment, among others.

Given the above, it is noteworthy that urban centers are factors of social, commercial and cultural interaction that attract people who come from all over the city, facilitating the commuting of patients and increasing the disease transmission. Therefore, it is understood that the geographical space, socially developed by men, incorporating social determinants in a vision of totality, is relevant for reorienting actions to monitor the conditions that generate the health-disease process in a place.

### Study limitations

The absence of some important information in secondary data collection, such as the address used in geocoding, resulted in a high number of unfilled fields. Furthermore, another limitation was the difficulty in contacting users due to contingency measures resulting from the COVID-19 pandemic.

### Contribution to health, nursing, or public policies

This study is pioneering in terms of spatial assessment, risk analysis and social care required for individuals affected by TB in the municipality of São Luís, Maranhão. Thus, in addition to knowing the chain of dissemination of TB transmissibility, through spatial analysis, it is essential to know what has been done in terms of social protection and individuals’ perception of this, which makes mixed studies of great importance.

## CONCLUSIONS

Awareness of areas at risk for developing the disease and SDH that are associated with TB makes it possible not only to effectively strengthen control strategies, but also to provide strategic information that improves several fundamental actions in combating this disease. Such actions include but are not limited to initiatives such as active search for cases, health education programs aimed at the population, effective notification process of new cases and identification of crucial factors for effective performance in primary care. This knowledge, when applied, not only enriches the epidemiological database, but also translates into more informed guidelines for implementing preventive and curative interventions, thus contributing to the overall effectiveness of public health efforts aimed at TB.
