# Supplementary material for: Social protection in areas vulnerable to tuberculosis: a mixed methods study in São Luís, Maranhão
Source: Rev Bras Enferm. 2024 Jun 17;77(3):e20230428. doi: 10.1590/0034-7167-2023-0428 (PMC11185072; doi:10.1590/0034-7167-2023-0428)
Supplement: Translation

# Translation: Proteção social em áreas vulneráveis à tuberculose: um estudo misto em São Luís, Maranhão

**DOI:** 10.1590/0034-7167-2023-0428pt

**Authors:** Francisca Bruna Arruda Aragão, Mellina Yamamura Calori, Thais Zamboni Berra, Antônio Carlos Vieira Ramos, Ethel Leonor Noia Maciel, José Henrique da Silva Cunha, Larissa Barros de Souza, Marcelino Santos, Ricardo Alexandre Arcêncio, Regina Célia Fiorati

**Affiliations:** I Universidade de São Paulo. Ribeirão Preto, São Paulo, Brasil; II Universidade Federal de São Carlos. São Carlos, São Paulo, Brasil; III Universidade Federal do Espírito Santo. Vitória, Espírito Santo, Brasil; IV Universidade Federal do Maranhão. Imperatriz, Maranhã, Brasil

**Keywords:** Determinantes Sociais da Saúde, Política Pública, Programa Social, Tuberculose, Vulnerabilidade Social

## Abstract

**Objetivos:**

analisar as áreas de risco para a tuberculose e as influências da proteção social no desenvolvimento do tratamento para a doença no município de São Luís, Maranhão.

**Métodos:**

trata-se de pesquisa de método misto sequencial explanatório. Na fase quantitativa, os dados foram obtidos do Sistema de Informação de Agravos de Notificação de 2010 a 2019, sendo realizado georreferenciamento para identificação das áreas vulneráveis à tuberculose. Na fase qualitativa, realizaram-se entrevistas semiestruturadas com indivíduos que recebiam benefício social.

**Resultados:**

foram geocodificados 7.381 casos, e, a partir da análise de varredura puramente espacial, foi possível identificar 13 aglomerados espaciais de risco. Quanto às entrevistas, verificou-se uma relação positiva entre a melhora dos pacientes e o recebimento de benefícios.

**Conclusões:**

o espaço geográfico e os determinantes sociais são relevantes para reorientação das ações de monitoramento das condições geradoras do processo saúde-doença.

## INTRODUÇÃO

Até o surgimento da COVID-19, doença causada pelo *Severe Acute Respiratory Syndrome Coronavirus 2* (SARS-CoV-2), a tuberculose (TB) era a doença infectocontagiosa que mais matava no mundo, entretanto, atualmente, ocupa o segundo lugar. Além disso, lidera os índices entre as doenças infectocontagiosas em países em desenvolvimento, afetando principalmente países e populações em situação de vulnerabilidade social. Estima-se que aproximadamente 1/3 da população está infectada pelo complexo do *Mycobacterium tuberculosis*, dessa maneira, dificultando ainda mais o controle da doença, principalmente em grupos e/ou populações que possuem maior risco de adoecimento, influenciados pelos condicionantes e Determinantes Sociais da Saúde (DSS)^(1)^.

O Brasil está na lista de 30 países que concentram 87% da carga da TB, ocupando a 20ª posição mundial, junto com outros 15 países, sendo responsável por 93% das notificações da TB no mundo, o que constitui um grande desafio para gestores e profissionais de saúde^(1)^. A doença se associa a determinantes sociais, políticos e culturais, bem como às condições de vida da população^(2)^, determinada por fatores biológicos, como desnutrição, infecção por *Human Immunodeficiency Virus* (HIV), faixa etária, e fatores sociais, como habitações insalubres, alta densidade demográfica, condições de trabalho inadequadas e falta de acesso aos serviços de saúde^(3)^.

Diante do exposto, a relação com os determinantes sociais, estruturais e intermediários, e políticas de proteção social é imprescindível para garantir melhoria das condições sociais e, assim, mais qualidade de vida das pessoas afetadas pela doença. Desse modo, no atual estudo, o embasamento teórico utilizado foi o Modelo dos DSS da Comissão dos DSS da Organização Mundial da Saúde (OMS)^(4)^. Nesse contexto, achados na literatura apontam elevação dos quadros de cura em países que agregam a abordagem do cuidado centrado na pessoa e oferecem suporte social à pessoa com TB e família, como vales transportes, cestas básicas e recursos financeiros^(5)^.

É válido destacar que estudos evidenciam queda na qualidade de vida da pessoa depois do início do tratamento em decorrência dos efeitos colaterais da medicação, sendo essencial o uso de estratégias farmacológicas e não farmacológicas para redução desses efeitos, e o suporte social faz toda a diferença nessa gestão da clínica^(5)^.

Uma outra questão se refere aos custos catastróficos, fazendo com que a pessoa com TB tenha muitas despesas financeiras com o tratamento, devido a idas frequentes aos serviços de saúde, interrupções dos dias de trabalho, gastos com transportes, despesas com alimentos fora de casa, entre outros^(3)^.

Dessa maneira, evidencia-se a necessidade de maior envolvimento entre os profissionais de saúde, gestores e comunidade científica, a fim de implementar medidas operacionais e de investigação que sejam relevantes para o conhecimento dos condicionantes sociais relacionados ao processo saúde-doença e as medidas e estratégias para mitigar a TB^(3)^.

Assim, na literatura, tem-se observado estudos intencionados a compreender a dinâmica da TB nos territórios, através do uso da geoepidemiologia, pelo Sistema de Informação Geográfica (SIG); todavia, ainda faltam estudos que evidenciem a cobertura de programas de proteção social dessas áreas.

Para tanto, observa-se a análise espacial como uma técnica de coleta e tratamento das informações em um determinado espaço geográfico. Quando utilizada na área da saúde, permite o mapeamento de agravos de saúde e avaliação de risco, contribuindo para a identificação de grupos vulneráveis, áreas de risco e da hierarquização de prioridades, subsidiando o desenvolvimento de intervenções e políticas públicas direcionadas e integrais em saúde.

## OBJETIVOS

Analisar as áreas de risco para a TB e as influências da proteção social no desenvolvimento do tratamento para a doença no município de São Luís, Maranhão.

## MÉTODOS

### Aspectos éticos

O estudo na fase quantitativa teve a dispensação do Termo Consentimento Livre e Esclarecido (TCLE), pois a busca dos dados foi realizada mediante o Sistema de Informação de Agravos de Notificação (SINAN), disponível em https://portalsinan.saude.gov.br/tuberculose de forma universal e integralizada. No entanto, na etapa qualitativa, o estudo foi conduzido de acordo com as diretrizes de ética nacionais e internacionais. Foi aprovado pelo Comitê de Ética em Pesquisa da Escola de Enfermagem de Ribeirão Preto (EERP). Dessa forma, o TCLE foi obtido de todos os indivíduos envolvidos no estudo por meio de documento escrito, sendo uma via entregue ao participante e a outra ao pesquisador. Para garantir o anonimato dos participantes, foi utilizada a letra E (entrevistado), seguida do número sequencial das entrevistas.

### Desenho do estudo

Trata-se de pesquisa de método misto, mediante a combinação de abordagens quantitativa e qualitativa, caracterizando-se pelo desenho sequencial explanatório. Trata-se de desenho explanatório porque a etapa quantitativa precede a qualitativa em duas fases distintas, porém interativas e sequenciadas^(6)^. O estudo segue as normas de acordo com *Preferred Reporting Items for Systematic Review and Meta-Analysis Protocols* (PRISMA). Foi realizado nos anos de 2010 a 2019 no município de São Luís, Maranhão, Brasil, com sete distritos sanitários de saúde.

### População e amostra

A população do estudo foi composta por indivíduos maiores de 18 anos, diagnosticados e em tratamento da TB e que recebiam algum benefício da Proteção Social identificadas nas áreas de risco.

Critérios de inclusão incluíram pcientes diagnosticados com TB que recebiam algum benefício social, federal, estadual ou local com a idade igual ou maior de 18 anos, residentes na zona urbana do município, cujo diagnóstico está descrito na Classificação Internacional de Doenças versão 10 – (CID10), com códigos correspondentes de A15.0 a A19.0. Critérios de exclusão incluíram notificações de casos diagnosticados que tiveram endereço em outros municípios do estado do Maranhão e/ou sem endereço completo na ficha de notificação, casos de repetição de digitação dos dados, idade inferior a 18 anos, pacientes que não estavam inseridos nos programas de benefício social.

Na etapa quantitativa, a coleta de dados foi realizada por meio da ficha de notificação do SINAN, que foi usada para mapear as áreas de riscos para a TB. A ficha deveria estar devidamente preenchida referente aos casos identificados com no mínimo um ano. Foram considerados, para critério de inclusão, casos de TB diagnosticados entre 01 de janeiro de 2010 e 31 de dezembro de 2019. Vale destacar que foram utilizados os dados das fichas de notificações do SINAN referentes aos anos de 2010 a 2019. Nesse período, o município de São Luís contava apenas com sete distritos sanitários de saúde.

### Análises dos resultados e estatística

Na etapa quantitativa, a coleta de dados foi realizada através da ficha do SINAN. Foram considerados os casos confirmados de TB, residentes em São Luís e, quando o acometido por TB foi notificado mais de uma vez, considerou-se apenas o registro mais atual.

Da ficha de notificação, foi coletado o endereço residencial de cada caso e, posteriormente, foi realizado o georreferenciamento, a partir das coordenadas geográficas (latitude e longitude) obtidas através da ferramenta *software Google Earth™*.

Para a detecção dos aglomerados de risco, aplicou-se a técnica de estatística de varredura puramente espacial^(7)^. Na análise, os aglomerados são identificados através de janelas circulares com raio variável ao redor do centroide de cada setor censitário do município em análise, onde é calculado o número esperado de casos dentro desse círculo. Assim, o Risco Relativo (RR) de cada aglomerado foi determinado, indicando a intensidade de ocorrência dos casos de TB concomitante no município analisado. Para a interpretação, quando o RR do conglomerado é igual a 1 (RR=1), há uma forte evidência que não há aglomerados; se menor do que 1 (RR<1), tendendo a zero, será de baixo risco (ou proteção); acima de 1 (RR>1), será área de risco. Foram considerados significativos os aglomerados com valor de p <0,05 (α=5%).

O georreferenciamento e a confecção dos mapas foram realizados a partir do *software* QGIS 3.16.2. A análise de varredura foi realizada a partir do *software* SaTScan™, versão 9.2. A caracterização dos entrevistados foi realizada a partir do *software* Stata, versão 13.0, e para a análise de conteúdo, foi utilizado o programa ATLAS, versão 9.

A fase qualitativa foi realizada a partir das áreas de risco identificadas na etapa anterior. Essa etapa foi conduzida a partir do *Consolidated criteria for Reporting Qualitative research* (COREQ), com acompanhamento dos resultados quantitativos que foram utilizados para refinar as questões qualitativas. Após, foram realizadas entrevistas norteadas por roteiros semiestruturados abordando categorias analíticas que trazem questões sobre saberes e práticas acerca da TB, recebimento de benefícios da assistência social, importância desses benefícios para paciente e sua influência no tratamento, direcionadas às pessoas diagnosticadas e identificadas a partir dos prontuários de pacientes que faziam tratamento nas Unidades Básicas de Saúde (UBS) e na Estratégia Saúde da Família (ESF), localizadas nas regiões de maiores riscos para TB e identificadas previamente na etapa quantitativa.

Destaca-se que, nessa etapa, foram entrevistados apenas os pacientes diagnosticados com TB que recebiam algum benefício social federal, estadual ou local. Para isso, foi construído um roteiro de entrevista realizada com pacientes portadores de TB em São Luís, Maranhão.

A partir da identificação dos potenciais participantes, essas pessoas foram contatadas pela pesquisadora para a apresentação do estudo. Após esse momento, realizou-se o refinamento das questões qualitativas que contavam no roteiro da entrevista.

Os dados dos prontuários e as declarações individuais foram mantidos em sigilo; as gravações foram feitas em áudio para manter as informações como realmente foram relatadas; e cada entrevista teve duração de aproximadamente uma hora, ficando à disposição do participante e mantida sob a responsabilidade e a guarda do pesquisador.

Vale ressaltar que as entrevistas foram realizadas entre os meses de agosto e outubro de 2021. A realização dessas obedeceu aos critérios como data e horário agendado. A partir da disponibilidade, a coleta dos depoimentos ocorreu nas dependências das UBS, em salas previamente reservadas. Destaca-se que uma das 16 entrevistas ocorreu na residência de um dos participantes que apresentava diagnóstico da TB e, por isso, possuía dificuldades de locomoção. Em virtude dessa dificuldade, visando ao bem-estar desse paciente, a pesquisadora se direcionou ao domicílio, acompanhada pela equipe de saúde e pelo agente comunitário para realização da entrevista.

Para a análise dos dados qualitativos, foi utilizada a técnica da análise de conteúdo^(8)^, desdobrada na pré-análise, seguida de leitura flutuante das entrevistas, permitindo à pesquisadora o aprofundamento no material coletado.

Em seguida, elas foram lidas exaustivamente, visando à compreensão profunda. Na fase de exploração do conteúdo, identificaram-se os núcleos de sentido, que foram agrupados sistematicamente, sendo analisados e reorganizados, dando origem a categorias analíticas.

## RESULTADOS

De acordo com os dados do SINAN, dos 7.958 casos de TB, notificados entre os anos de 2010 e 2019, foram geocodificados 7.381 (92,7%). De acordo com o perfil sociodemográfico, a maioria representou indivíduos do sexo masculino (67,92%) com faixa etária de 20–29 anos (27,07%) e 30–39 anos (23,67%). A maior parcela dos casos se declarou parda (50,11%), possuindo de um a oito anos de estudos (47,40%). Uma pequena parcela alegou receber algum tipo de auxílio governamental (4,39%) (Tabela 1). A Figura 1 representa os casos distribuídos no território espacial do município, em que se nota distribuição heterogênea dos casos.

**Tabela 1:** Caracterização dos aglomerados espaciais de risco de casos de tuberculose em São Luís, Maranhão, Brasil, de 2010 a 2019

Identificação	Número de setores	Total de casos	População
1 Centro de Saúde de Pedrinhas I	1	556	1.300
2 USF Vila Sarney, Centro de Saúde Thales Ribeiro, Centro de Saúde Tibiri	1	25	220
3 Centro de Saúde Fabriciana Moraes	1	61	1.525
4 USF Olímpica III	2	13	388
5 USF Vila Sarney	2	101	3.622
6 USF Santa Efigênia	1	33	1.183
7 Centro de Saúde Radional	1	26	992
8 Centro de Saúde São Raimundo	1	25	1.042
9 USF Olímpica com USF Olímpica II na proximidade	1	25	1.222
10 Centro de Saúde Yves Parga, Centro de Saúde Janaina, Centro de Saúde Laura Vasconcelos, Centro de Saúde Dr. Paulo Ramos e Centro de Saúde da Liberdade	94	924	78.226
11 Centro de Saúde Bairro de Fátima, Centro de Saúde do João Paulo, Centro de Saúde Dr. José Carlos Macieira, Centro de Saúde Especialidades Médicas da Vila Esperança, Centro de Saúde Antônio Guanare, Centro de Saúde Genésio Rêgo, Centro de Saúde Vila Embratel, Centro de Saúde Vila Bacanga Embrião e Centro de Saúde Itapera	217	2.773	27.727
12 Centro de Saúde Bairro de Fátima	2	852	79.219
13 Centro de Saúde Gapara com Centro de Saúde da Vila Embratel na proximidade	2	474	44.970

**Figura 1:** Distribuição espacial dos casos de tuberculose residentes em São Luís, Maranhão, Brasil, entre 2010 e 2019

A distribuição, embora heterogênea, concentra-se na faixa litorânea e no extremo norte e noroeste da região. Na análise de varredura puramente espacial, foi possível identificar 13 aglomerados espaciais de risco para TB, apresentados na Tabela 1.

A Figura 2 apresenta as áreas de risco identificadas, com a análise de varredura puramente espacial, segundo setores censitários de São Luís, Maranhão.

**Figura 2:** Áreas de risco espacial para tuberculose segundo setores censitários, ponto de localização das unidades de saúde e risco relativo de cada aglomerado

### Etapa qualitativa

Essa etapa foi realizada com pacientes oriundos do aglomerado 11, devido ao maior número de centros de saúde disponíveis. Dessa forma, foi utilizada a técnica de análise de conteúdo de Bardin, em que foram entrevistadas 16 pessoas, sendo que 20 pessoas não referiram recebimento de nenhum auxílio social. Após análise de conteúdo, as entrevistas foram divididas em três principais categorias analíticas.

Em relação ao “Recebimento de benefícios da assistência social”, verificou-se que a maioria recebia algum benefício da assistência social antes do diagnóstico da doença. Entre os auxílios citados, estão a aposentadoria, considerado como benefício da assistencial social, concomitantemente ao Bolsa Família, todavia observaram-se relatos quanto ao recebimento do auxílio emergencial como ajuda no sustento de sua família.

*Eu recebo Bolsa Família, mas agora estou recebendo o auxílio emergencial.* (E9)

*Eu recebo R$89,00 por mês do Bolsa Família, inclusive fiz meu cadastro lá no* [posto] *no tempo que eu estava em situação de vulnerabilidade. Estava desempregado, sem moradia, por isso recebi esse auxílio do Governo Federal.* (E10).

*Eu recebo minha aposentadoria, é pouco, mas nos ajuda aqui em casa. Minha aposentadoria já me ajudou, porque eu comprei uma moto e paguei só com esse dinheiro. Não tirei dinheiro do meu negócio. Ela me ajuda.* (E14)

[...] *porque recebo o Bolsa Família aqui, então o Bolsa Família deve aumentar um pouco.* (E16).

Na categoria analítica “Importância do benefício”, verifica-se que as narrativas das vidas desses pacientes antes do recebimento do benefício estão voltadas para as dificuldades das pessoas em relação à criação dos filhos, à alimentação, à moradia, à educação e à saúde.

*Antes do benefício, tinha somente meu salário, que é pouco* [...] *então, em alguns meses, faltavam coisas para casa ou para as crianças.* (E6)

*Era difícil antes, pois meu marido está desempregado, e eu preciso pagar transporte, eu preciso me* [...] *alimentar também, já que a alimentação é um pouco precária.* (E8)

*Moramos cinco pessoas aqui em casa, e somente meu marido quem trabalha, então havia dias em que não tínhamos muito o que comer, além de não poder comprar alguns itens básicos para as crianças, como um sapato, por exemplo*. (E10)

*Ah, se não recebesse o bolsa, não daria para viver bem e faltaria um monte de coisas para meus filhos*. (E15)

*Eu acho que devemos receber, porque não podemos trabalhar, então como vamos nos manter?* (E5)

Em relação à categoria analítica “O benefício e sua influência no tratamento de tuberculose”, encontraram-se associações positivas dos programas beneficiários sociais com a duração do tratamento, uma vez que seus efeitos diretos estão relacionados à redução do abandono ao tratamento devido à possibilidade da compra de medicamentos, que está associada ao aumento da cura e, consequentemente, da redução de óbitos relacionados à doença, além da aquisição de alimentos para eles e para os familiares.

*Sim, tanto para mim quanto para outras pessoas. Quando estamos passando por dificuldades e não temos nem dinheiro para comprar um remédio, nos ajuda muito.* (E1)

*Ajuda muito, ajuda sim, preciso me alimentar bem, preciso de uma alimentação normal para eu poder ganhar peso* [...] *já ganhei bastante! Antes, o meu peso não estava normal, estava muito baixo.* (E3)

*Tem sim,* [...] *como não estou trabalhando, esse dinheiro me ajuda a comprar alimentos e frutas.* (E4)

*Creio que sim,* [...] *na atual crise que estamos passando, existem os problemas de alimentação* [...] *eu cortei alguns alimentos* [...] *eu consigo me alimentar bem, mas tem uns pacientes que precisam comprar o alimento para complementar o tratamento, e esse auxílio poderia melhorar a recuperação.* (E12)

Entretanto, observou-se que três pacientes relataram que o benefício não produziu nenhuma mudança, visto que acham pouco o valor adquirido.

*Não dá para comprar muitas coisas, mas* [...] *já ajuda um pouco, dá pra levar.* (E2)

*Não, porque, como eu faço um bico aqui, outro ali, não dependo só desse dinheiro, porque, com esse dinheiro, não consigo fazer tudo que eu quero, entendeu?* (E9)

*Não, não influenciou muito, não, porque R$89 é muito pouco.* (E10)

## DISCUSSÃO

Na presente pesquisa, foi realizada a identificação das áreas de risco relacionadas à TB, adentrando uma análise aprofundada sobre como as políticas de proteção social incidem no avanço do tratamento dessa enfermidade no município de São Luís, Maranhão, onde a maioria representou indivíduos do sexo masculino com faixa etária de 20–29 anos e 30–39 anos, pardos e com um a oito anos de estudos. Uma parcela alegou receber algum tipo de auxílio governamental.

Silva *et al*.^(9)^, em uma análise epidemiológica da correlação entre fatores sociodemográficos e a incidência da TB na região Nordeste, verificaram que a doença acomete mais homens (63,2%), pardos (60,2%) e com faixa etária entre 20 e 64 anos (70,6%), corroborando os achados.

Da mesma forma, o estudo de Oliveira *et al*.^(10)^, analisando o perfil da TB na região Nordeste do Brasil durante o período de 2008 a 2018, observou que a doença apresentou maior incidência entre indivíduos de 20 a 29 anos, sendo a maioria no sexo masculino (38.306 casos).

Refere-se ainda que a escolaridade é um DSS, uma vez que a educação influencia na capacidade do indivíduo em adquirir informações de promoção da saúde, prevenindo-se doenças e ampliando a adesão ao tratamento^(11)^.

No período de 2010 a 2019, foram identificados 13 aglomerados espaciais que apresentam alto risco para ocorrência da TB em São Luís. Os aglomerados identificados são espaços favoráveis a apresentar as maiores taxas da doença, devido às características como maior concentração populacional e fluxo intenso de pessoas vindo de diversas localidades.

De acordo com Silva *et al*.^(9)^, o maior número de casos de TB em regiões menos favorecidas é reflexo da falta de ações ligadas à busca de sintomáticos respiratórios para detecção precoce e, também, por menor procura dos moradores pelos serviços de saúde em regiões periféricas, assim como pela acentuada aglomeração populacional na região, resultando no maior número de casos de TB e elevada incidência. Em vista disso, esse cenário demonstra a desigualdade social no Brasil, já que o acesso aos recursos de saúde, educação, distribuição de renda e saneamento básico está intimamente relacionado ao risco de adoecer por TB em diversas camadas sociais.

Na pesquisa de Asemahagn, Alene e Yimer^(12)^, realizada na Etiópia, verificaram que a maioria dos aglomerados de casos de TB foi detectada nas áreas central (urbana e seus arredores), norte e noroeste da zona. Os autores ressaltam que essas áreas possuem grande cobertura geográfica com características desfavoráveis (vales, infraestruturas montanhosas e limitadas de transporte), alta densidade populacional com baixa renda, acesso limitado às unidades de saúde e pouca conscientização da comunidade sobre a prevenção da TB.

Considera-se, portanto, que o espaço do município estudado é heterogêneo e pode apresentar maior variabilidade na distribuição dos indicadores de saúde e, por consequência, nas taxas de TB. Deve-se também destacar que a enfermidade pode estar preponderantemente relacionada à pobreza, corroborando a relação doença negligenciada *versus* DSS.

Pelas narrativas sobre a situação em que viviam essas famílias no período anterior ao benefício, é possível observar os múltiplos problemas por elas enfrentados e que representam obstáculos à sua autonomia como cidadãs. Tal situação vai de encontro com os da DSS da OMS, visto que os entrevistados apresentam vulnerabilidades e iniquidades sociais, produzindo acessos diferenciados a recursos materiais e simbólicos que impactam na saúde de pessoas e populações.

De acordo com o estudo de Alves *et al*.^(13)^, a pobreza foi um determinante social fundamental para explicar o conglomerado de risco de morte por TB. Os autores afirmam que um determinante social importante se refere à renda familiar, dado que os resultados indicam que o aumento desse fator diminui o risco de morte por TB, ou seja, as áreas de maior renda apresentam melhores níveis de saúde geral e pouca mortalidade pela doença. Assim, baixa renda e pobreza são indicadores que se relacionam, embora não se restrinjam exclusivamente a esse quesito.

Os serviços de saúde públicos são fundamentais para o controle da TB, contudo são insuficientes no país. Melhorias nos serviços e programas de controle da TB podem acarretar melhorias no atendimento, com mais rapidez e eficácia para o diagnóstico e manejo dos casos de TB.

Além disso, encontrar e tratar as pessoas mais pobres ainda é o maior desafio, visto que o foco na população vulnerável determina múltiplas abordagens que geralmente não estão sob a competência padrão dos profissionais de saúde no Brasil^(14)^.

Nesse contexto, observa-se a importância dos benefícios provenientes de programas do sistema de proteção social para essa população, ficando evidente no decorrer do estudo que a maioria considera essencial para sua sobrevivência e para o tratamento da TB.

Orlandi *et al*.^(15)^ afirmam que o Brasil disponibiliza o tratamento da TB gratuitamente no Sistema Único de Saúde (SUS), mas existem evidências de que os pacientes apresentam despesas proeminentes com a saúde mesmo indiretamente, a exemplo da necessidade de transporte para se dirigir ao serviço de saúde e de alimentação. No caso da TB, estima-se que esses gastos comprometem cerca de 8% a 20% do rendimento anual dos doentes. Portanto, o apoio social, com a oferta de alimentos, vale-transporte ou apoio monetário, pode diminuir os efeitos da enfermidade e auxiliar na superação de algumas barreiras do tratamento. Na pesquisa dos autores supracitados, observa-se que o recebimento da cesta básica e do vale-transporte foi relevante para a adesão ao tratamento, assim como para o vínculo entre o paciente e a equipe de saúde.

De acordo com os participantes do estudo que estavam inseridos nas regiões que compõem os aglomerados de risco para a TB, identificou-se que os benefícios sociais, como o antigo Bolsa Família, atualmente denominado Auxílio Brasil, e o auxílio emergencial, foram ressaltados como importantes garantias para o enfrentamento do desemprego, sobretudo durante a pandemia de COVID-19.

Oliosi *et al*.^(16)^ observaram que a taxa de cura é 7,6% maior no grupo que recebia o Bolsa Família em comparação ao grupo que não recebia, e a proporção de pacientes que desistiram foi 7% menor no grupo que dos que recebiam o benefício. De acordo com os autores, o Bolsa Família possui efeito direto no resultado do tratamento da TB. Isso está de acordo com o estudo de Carter *et al*.^(17)^ que demonstrou um aumento absoluto substancial na taxa de sucesso do tratamento da TB (7% e 11%) entre os que recebem Bolsa Família.

Cumpre lembrar que ocorreu grande impacto da COVID-19 no tratamento da TB, uma vez que ocasionou desafios adicionais para além da vigilância epidemiológica, na esfera nacional e internacional, e no reconhecimento da necessidade de políticas públicas voltadas à diminuição das desigualdades de acesso aos sistemas de saúde e da urgente redução da injustiça social^(18)^.

De acordo com Maciel *et al*.^(18)^, a COVID-19 e a TB são doenças diferentes, com impactos distintos na saúde pública, estabelecendo ações diferenciadas dos governos para seu enfrentamento, sem investimentos corretos para o controle e inovação para ambas as doenças. No Brasil, o financiamento para a TB não atingiu 0,1% do valor total alocado para a ciência e tecnologia em todas as áreas na época da pandemia. De acordo com o Portal de Transparência do governo brasileiro, o valor total investido nos últimos dez anos na pesquisa da TB foi de pouco mais de US$ 6 milhões, valor que foi ainda mais reduzido durante o período de pandemia^(19)^.

Torres e Rabahi^(20)^ afirmam que outro impacto imediato da pandemia de COVID-19 foi a redução de novos diagnósticos. Ou seja, o aumento de notificações da TB mundialmente entre os anos de 2017 e 2019 foi seguido de uma diminuição de 18% no intervalo entre 2019 e 2020 (de 7,1 milhões para 5,8 milhões), no qual o Brasil está entre os países que mais contribuíram para essa diminuição. Concomitante a esse panorama, ocorreu uma ampliação de óbitos por TB nas áreas global, regional e por países, revertendo anos de progresso na redução do número de mortes pela doença^(21)^.

Esse cenário também é demonstrado, conforme Migliori *et al*.^(22)^, através da diminuição dos testes de TB em alguns dos países incluídos no estudo, como as Filipinas, o Quênia e o Brasil. As quedas relativas à terapia preventiva foram de 30% a 70% em vários centros de TB no Brasil, no Quênia, nas Filipinas e na Rússia. Os autores ressaltam ainda que, para conter a disseminação do novo coronavírus, a sociedade foi orientada a ficar em casa. Porém, essa política se tornou inviável em alguns aspectos, já que, em alguns países em desenvolvimento, os trabalhos informais são a maioria: 54% na América Latina; 67% no Sudeste Asiático; e 86% na África.

Logo, esses trabalhadores não puderam ter a opção de ficar em seu domicílio e nem todos os governos puderam fornecer assistência financeira de emergência, o que possibilitaria a permanência dessas pessoas em casa. Quanto mais elevado o nível de pobreza, maior o número de casos de COVID-19, o que, consequentemente, gerou uma diminuição nos recursos e na conscientização direcionados a outras doenças, como a TB^(23)^.

Horta *et al*.^(24)^, através de um estudo com 133 cidades brasileiras no período da pandemia, demonstraram que, dos 33.250 indivíduos pesquisados, 11,8% relataram que deixaram de procurar atendimento, apesar de estarem doentes, 17,3% não compareceram às consultas de rotina ou triagem e 23,9% relataram um ou ambos os desfechos. O estudo demonstrou também que os indivíduos com menor nível socioeconômico e escolaridade tinham maior probabilidade de não procurar serviços de saúde em caso de TB. Souza *et al*.^(25)^, a partir disto, afirmaram que os DSS devem ser compreendidos além de seus aspectos biomédicos, incorporando subjetividade do acesso, vínculo, acolhimento, necessidades de saúde, adesão ao tratamento, entre outros.

Diante do exposto, ressalta-se que os centros urbanos são fatores de interação social, comercial e cultural que atraem pessoas que vêm de todos os lugares da cidade, facilitando a circulação de doentes aumento da transmissão da doença. Logo, entende-se que o espaço geográfico, socialmente desenvolvido pelos homens, incorporando os determinantes sociais em uma visão de totalidade, é relevante para reorientação das ações de monitoramento das condições geradoras do processo saúde-doença de um lugar.

### Limitações do estudo

A ausência de algumas informações importantes na coleta de dados secundários, como o endereço utilizado na geocodificação, ocasionou elevada quantidade de campos não preenchidos. Além disso, outra limitação foi a dificuldade de contato com usuários devido às medidas de contingência resultantes da pandemia de COVID-19.

### Contribuição para as áreas da saúde, enfermagem e políticas públicas

Este estudo é pioneiro no que se refere à avaliação espacial, análise de risco e de atenção social demandada aos indivíduos afetados por TB no município de São Luís, Maranhão. Assim, além de conhecer a cadeia de disseminação da transmissibilidade da TB, por meio da análise espacial, é essencial conhecer o que tem sido realizado em termos de proteção social e a percepção dos indivíduos quanto isto, o que torna de grande importância os estudos mistos.

## CONCLUSÕES

O conhecimento das áreas de risco para o acometimento da doença e os DSS que são associados à TB possibilita não apenas o fortalecimento eficaz das estratégias de controle, mas também a disponibilização de informações estratégicas que aprimoram diversas ações fundamentais no enfrentamento dessa enfermidade. Tais ações incluem, mas não se limitam a, iniciativas como a busca ativa por casos, programas de educação em saúde direcionados à população, o efetivo processo de notificação de novos casos e a identificação de fatores cruciais para uma atuação eficaz na atenção primária. Esse conhecimento, ao ser aplicado, não apenas enriquece a base de dados epidemiológica, mas também se traduz em diretrizes mais embasadas para a implementação de intervenções preventivas e curativas, contribuindo, assim, para a eficácia global dos esforços de saúde pública voltados para a TB.
